# Response to Birth Weight and Renal Functional Reserve in Adults

**DOI:** 10.1016/j.ekir.2023.06.012

**Published:** 2023-06-17

**Authors:** Bjørn Steinar Lillås, Camilla Tøndel, Toralf Melsom, Bjørn Odvar Eriksen, Hans-Peter Marti, Bjørn Egil Vikse

**Affiliations:** 1 Department of Medicine, Haugesund Hospital, Haugesund, Norway; 2 Department of Clinical Medicine, University of Bergen, Bergen, Norway; 3 Department of Pediatrics, Haukeland University Hospital, Bergen, Norway; 4 Metabolic and Renal Research group, UiT The Arctic University of Norway, Tromsø, Norway; 5 Section of Nephrology, University Hospital of North Norway, Tromsø, Norway; 6 Department of Medicine, Haukeland University Hospital, Bergen, Norway

The author Replies:

In this issue Tsuboi and Bertram^1^ have commented on our paper “Renal functional response–association with birth weight and kidney volume.”S1 We would like to thank the authors for their appreciation of our research and for questioning the level of prematurity of our low birth weight (LBW) cohort. It is argued that being born close to term, yet premature, the kidneys of the LBW group may have been too well developed to allow for a reduced renal functional response. This is a valid argument that unfortunately was not discussed in our paper. However, we did not find any association between gestational age and renal functional response , and with a range of gestational age in the LBW group from 27 to 43 weeks, with a median of 34 weeks, this should have been picked up. Using the same cohort, we have previously shown that women born LBW had lower measured glomerular filtration rate and lower kidney volume than their normal birth weight counterparts.S2^,^^2^ In these papers, both LBW and small for gestational age were important variables, whereas gestational age was not. This suggests an important contribution of the birth weight alone, regardless of the gestational age. LBW is commonly used as an umbrella including both fetal growth restriction, prematurity, and small for gestational age.^3^ Premature birth, and especially very premature birth, disrupts the natural nephrogenesis.^4^ Although, there is evidence of postnatal nephrogenesis in premature children, this is both qualitatively and quantitatively insufficient to merit a normal nephron endowment.S4 Therefore, prematurity is an important determinant of nephron number. Other studies have shown reduced nephron number and impaired kidney health later in life associated with LBW and small for gestational age both with and without prematurity.^2^^,^^5^^,^^6^ A Swedish registry study showed not only an increased risk of chronic kidney disease of both extremely early and late preterm, but also for those born early term compared to full term.^6^ Therefore, intrauterine growth restriction as evidenced by either LBW or small for gestational age may be as important determinants of nephron endowment as prematurity.

## Supplementary Material

Supplementary File (PDF)

**Supplemental References.**
