# Supplementary material for: Response to Birth Weight and Renal Functional Reserve in Adults
Source: Kidney Int Rep. 2023 Jun 17;8(8):1700–1. doi: 10.1016/j.ekir.2023.06.012 (PMC10403668; doi:10.1016/j.ekir.2023.06.012)
Supplement: Supplementary File (PDF) [file mmc1.pdf]

Supplemental references

S1. Lillas BS, Tøndel C, Melsom T*, et al.* Renal Functional Response-Association With Birth Weight and Kidney Volume. *Kidney international reports* 2023; **8:** 1034-1042.

S2. Lillås BS, Tøndel C, Aßmus J*, et al.* Low birthweight is associated with lower glomerular filtration rate in middle-aged mainly healthy women. *Nephrology, dialysis, transplantation : official publication of the European Dialysis and Transplant Association - European Renal Association* 2020; **37:** 92-99.

S3. Lillås BS, Qvale TH, Richter BK*, et al.* Birth Weight Is Associated With Kidney Size in Middle-Aged Women. *Kidney international reports* 2021; **6:** 2794-2802.

S4. Rodriguez MM, Gomez AH, Abitbol CL*, et al.* Histomorphometric analysis of postnatal glomerulogenesis in extremely preterm infants. *Pediatr Dev Pathol* 2004; **7:** 17-25.

S5. Gjerde A, Lillås BS, Marti HP*, et al.* Intrauterine growth restriction, preterm birth and risk of end-stage renal disease during the first 50 years of life. *Nephrology, dialysis, transplantation : official publication of the European Dialysis and Transplant Association - European Renal Association* 2020; **35:** 1157-1163.

Følsomhet Intern (gul)
